# The economic burden of diabetes in India: a review of the literature

**DOI:** 10.1186/s12992-014-0080-x

**Published:** 2014-12-02

**Authors:** Charles AK Yesudian, Mari Grepstad, Erica Visintin, Alessandra Ferrario

**Affiliations:** School of Health Systems Studies, Tata Institute of Social Sciences, Mumbai, India; LSE Health, London School of Economics and Political Science, Houghton Street, London, WC2A 2AE UK; Social Policy Department, London School of Economics and Political Science, Houghton Street, London, WC2A 2AE UK

**Keywords:** India, Diabetes, Cost of illness, Economic burden, Out-of-pocket expenditure

## Abstract

**Background:**

Diabetes and its complications are a major cause of morbidity and mortality in India, and the prevalence of type 2 diabetes is on the rise. This calls for an assessment of the economic burden of the disease.

**Objective:**

To conduct a critical review of the literature on cost of illness studies of diabetes and its complications in India.

**Methods:**

A comprehensive literature review addressing the study objective was conducted. An extraction table and a scoring system to assess the quality of the studies reviewed were developed.

**Results:**

A total of nineteen articles from different regions of India met the study inclusion criteria. The third party payer perspective was the most common study design (17 articles) while fewer articles (n =2) reported on costs from a health system or societal perspective. All the articles included direct costs and only a few (n =4) provided estimates for indirect costs based on income loss for patients and carers. Drug costs proved to be a significant cost component in several studies (n =12). While middle and high-income groups had higher expenditure in absolute terms, costs constituted a higher proportion of income for the poor. The economic burden was highest among urban groups. The overall quality of the studies is low due to a number of methodological weaknesses. The most frequent epidemiological approach employed was the prevalence-based one (n =18) while costs were mainly estimated using a bottom up approach (n =15).

**Conclusion:**

The body of literature on the costs of diabetes and its complications in India provides a fragmented picture that has mostly concentrated on the direct costs borne by individuals rather than the healthcare system. There is a need to develop a robust methodology to perform methodologically rigorous and transparent cost of illness studies to inform policy decisions.

## Background

Diabetes is one of the leading causes of morbidity and mortality worldwide [1-3] and a major problem in India. In 2012, 60% of all deaths in India were due to non-communicable diseases (NCDs), including cardiovascular diseases (26%), chronic respiratory diseases (13%), cancer (7%), diabetes (2%) and other NCDs (12%) [4,5]. Currently accounting for 43% of total disability adjusted life years (DALYs), the prevalence of NCDs is expected to increase in the coming years due to ongoing large-scale urbanisation and increasing life expectancy [3].

The prevalence of diabetes in 2013 in India is only slightly higher than the world average (9.1% vs. 8.3% worldwide) [3]. However, due to its very large population, India has the world’s largest population living with diabetes after China. In 2013, there were 65.1 million people between 20 and 79 years of age with diabetes and this number was predicted to rise to 109 million by 2035. The growing epidemic of type 2 diabetes in India has been highlighted in several studies [6-9].

Studies have shown large regional and socioeconomic differences in the prevalence of type 2 diabetes in India. Self-reported prevalence is lower in rural areas than in urban areas ranging from 3.1% in rural areas to 7.3% in urban areas [10]. The disease appears to be more prevalent in the south of the country as compared to the northern and eastern parts [11]. However, the absence of large well-planned national studies on diabetes prevalence have led to incomplete and unreliable nationwide data on the prevalence of diabetes in India [6].

Financing and delivery of health care in India has been left largely to the private sector [12]. In 2012, public health care funding was lower in India than other countries in the region, with a general government funding for health accounting for 33% of total health expenditure in India compared to an average of 52% in the South East Asia region [13]. Nevertheless, at 4% of India’s gross domestic product (GDP) the share of health expenditure is equivalent to the average of the South East Asia region [14].

At the 56^th^ World Health Assembly in Geneva in 2012, universal health coverage was identified as essential to consolidate public health advances [15]. While various health programmes and policies have previously attempted to achieve universal health coverage in India, there is still a long way to go. In 2010, only about 19 percent of the population (240 million people) was covered by the country’s central and state government-sponsored health insurance [16]. When including private insurance and other schemes, some 25 percent of the population (300 million people) was covered [16]. Thus, the financial burden of health care falls heavily on individuals with the government contributing to one third of total health spending and out-of-pocket payments representing about 58% of total health spend in 2012 [13].

The assessment of the economic and social impact of diabetes in India is important for several reasons. First, India is considered the diabetes capital of the world [17], yet not enough is done to tackle the disease. An article published in 2007 suggests that an estimated USD 2.2 billion would be needed to sufficiently treat all cases of type 2 diabetes in India [18]. In comparison, health spending per capita in 2012 was USD 61 [19]. Second, by 2025, most people with diabetes in developing countries will be in the 45 to 64 year age group, thus threatening the economic productivity of the country and the income-earning ability of individuals [20]. Third, the management of diabetes and its complications can be expensive, which poses serious obstacles to the strengthening of the Indian health care system and the Government’s plan to achieve universal health coverage by 2022.

As the burden of diabetes on total health care spending is likely to increase and, potentially, will have important consequences on the sustainability of health care financing, this study presents a critical review of the literature on cost of illness of diabetes and its complications in India and also makes recommendations on areas requiring further attention and research.

## Methods

A comprehensive literature review of the direct and indirect costs of diabetes in India was conducted in October 2014 following the Preferred Reporting Items for Systematic Reviews and Meta-Analyses (PRISMA) [21] guidelines.

### Search strategy

Searches were performed for all papers published up to 18 October 2014 in relevant databases (PubMed, Web of Science and Scopus). Reference lists in the articles included in the review were searched to identify further eligible articles.

### Search terms

Search terms and their combinations are presented in Table 1. Databases were searched using the primary term “India” in combination with one term associated with diabetes and complications from diabetes (column 2, Table 1) and one term associated with costs (column 3, Table 1).

**Table 1:** **Search terms**

	***Combined with (individually)***	***Combined with (individually)***
India	diabetes	expenditure
	diabetic	expenses
	“diabetic complications”	cost
	neuropathy	“economic burden”
	nephropathy	
	“renal replacement”	
	“chronic kidney”	
	“diabetic foot”	
	“diabetic ulcer”	

### Inclusion criteria

Papers were included if they provided original research findings on the cost (direct and indirect) of diabetes and its complications in India, were written in English and met the inclusion criteria following the PICOS approach, adapted to meet the needs of the review [22]. We did not include cost-benefit, cost-effectiveness, cost-minimisation and cost-utility analyses. The population considered consisted of people diagnosed with type 1 or 2; the contexts of interest were hospitals, clinics, and home settings in India, outcomes comprised direct and indirect costs for health systems, households and individuals; and, the relevant study designs were randomised controlled trials (RCTs), cohort and observational studies and surveys.

### Critical review of the data and quality of the studies

The review included articles reporting on the economic burden of diabetes using both quantitative and qualitative methods to elicit information on costs. In conducting our analysis, we have developed two extraction tables in two different Excel spreadsheets [23] in which the data was summarised. In the first one, we used predefined categories such as the year published, the research objectives, the methods and the sample characteristics for each article. Relevant findings were classified using a framework developed to guide the analysis of retrieved cost data (Table 2). Historical conversion rates from www.xe.com/currencytables/ were applied to report on cost estimates in both INR and USD throughout the article.

**Table 2:** **Classification of costs and economic impact on individuals and society**

**Economic impact on individual and household**		**Economic impact on health sector and economic sector**	
*Direct costs*	Hospital, transport, drug costs, foods	*Direct costs (health sector)*	Inpatient care, outpatient care (GPs, district hospitals, pharmacy), long-term care
*Indirect costs*	Loss of income associated with morbidity, mortality and disability	*Indirect costs (economic sector)*	Costs due to absenteeism, permanent disability and mortality

In the second spreadsheet we listed a number of technical criteria for a sound cost of illness study (COI). The quality indicators were selected based on criteria proposed by previous reviews and good practice guidelines [24-27] and adjusted in accordance with specific features of diabetes. Following data extraction, a score of either 0, 0.5 or 1 was assigned for each quality indicator. This led to a maximum obtainable score of 17.

An indicator was assigned the score of 1 if the quality and the appropriateness of the parameter were high, a score of 0.5 was assigned in the case quality parameter was only partially met and a score of 0 was assigned if there was no information on the particular parameter (unless a logical reason justifying the lack of this information was provided).

All the details of the parameters employed are presented in Table 3.

**Table 3:** **Quality indicators for cost of illness studies**

**General**	
Objective of the study	**Cost of drugs:** studies that aim to calculate the cost of a specific drug
	**General Costs:** studies that aim to calculate direct or indirect costs for the diabetes in general, for ambulatory care or for a specific subgroup.
	**Cost of Complication:** studies that aim to calculate the cost of a specific complication of diabetes.
How is the disease defined?	Diabetes type 1/Diabetes type 2/ Gestational diabetes
Is the definition clear and precise?	1 = the definition of the type of diabetes considered is clear and all the morbidities and co-morbidities considered are listed. 0 = the definition is vague and do not include any details of all the morbidities and co-morbidities considered
Which complications the authors have included?	1 = more than 4 complications are considered and specified.
	0,5 = up to 3 complications are considered for each patient but they are not specified.
	0 = no complications are considered or if they are considered there is no clear documentation in their inclusion.
Is a clear epidemiological definition provided?	The type of diabetes studied is specified
**SAMPLE**	
Which is the population sample considered?	Description of the population considered by the study.
Is the population selected appropriate?	The sample size is sufficiently large and the epidemiological characteristics of the population are in line with the objectives of the study. For example, a large national assessment of diabetes requires a large sample with a balanced population composition in terms of social class, the gender and other factors such as the education level. For a study focused on the costs of drug an appropriate sample could be small but should be focused on a particular health history of patients.
Are sources for population data reliable?	1 = self-assessment and questionnaire are confirmed by hospital records or hospitals and practitioners’ bills.
	0,5 = The only sources of data are questionnaire and self-assessment.
	0 = The sources of data are not defined or are subject to a number of biases.
The period of evaluation is appropriate?	A period of evaluation is considered appropriate if is equal or more than 6 months for prevalence- based studies and consider more than 1 year for incidence based studies.
**COSTS**	
Direct costs:	All resource costs employed to treat patients with diabetes (care and/or assistance). It includes medical and non-medical costs.
Indirect costs:	All the costs associated with the loss of productivity resulting from morbidity and mortality caused by diabetes.
Intangible costs:	All the costs associated with all the negative effects caused by the disease leading to deterioration in the quality of life of patients (e.g. isolation, anxiety, pain).
**Healthcare costs**	
People with the health condition	Premiums and levies paid to collectively financed healthcare systems; out-of-pocket costs of healthcare services and products; transport costs related to treatment; home and car modifications; special diets; domestic care; lost income for unpaid leave to attend treatment.
Others, including family members	Premiums and levies paid to collectively financed healthcare systems; out-of-pocket costs of healthcare and domestic services and products and home and car modifications for sick family members.
Healthcare system (public and private)	Hospitals; primary care services; nursing homes; pharmaceuticals; domiciliary care; rehabilitation; home nursing; medical specialists; general practitioners; community healthcare services; ambulance services; paramedical services; specialist equipment; diagnostic tests; training; research; infrastructure; equipment; preventive programmes; administration
Business/industry/employers (includes government employers)	Premiums and levies paid to collectively financed healthcare systems; preventive programmes
Government (excluding health care system)	Specialist equipment/infrastructure modifications; community support services; residential support services; preventive programmes (e.g. education and training)
**Other resource use**	
People with the health condition	Legal representation; childcare
Others, including family members	Damage to property (e.g. for substance abuse, smoking), crime-related costs (e.g. for substance abuse)
Healthcare system (public and private)	None
Business/industry/employers (includes government employers)	Worker replacement costs (recruitment, training, retraining); cost of implementing and adhering to regulation and legislation
Government (excluding health care system)	Regulation, inspection and monitoring; child welfare services; disability support services; courts services; police services; prison services; emergency/fire services; cost of administering additional taxes, levies and benefits.
**Production losses**	
People with the health condition	Lost income due to unpaid sick leave (absenteeism), treatment related time off work, temporary unemployment, reduced on-the job productivity (‘presenteeism’), premature retirement through morbidity or early mortality, unwanted job changes, loss of opportunities for promotion and education; loss of unpaid production while ill.
Others, including family members	Loss of income and unpaid production while caring for sick family members and friends.
Healthcare system (public and private)	None
Business/industry/employers (includes government employers)	Lost paid and unpaid output due to sickness (absenteeism for paid output), treatment-related time off work, temporary unemployment, reduced capacity, reduced on-the job productivity (‘presenteeism’), work injury, premature retirement through morbidity or early death
Government (excluding health care system)	None
**Intangible costs**	
People with the health condition	Quality of life (health, functioning, psychosocial impacts, including loss of leisure time), premature loss of life
Others, including family members	Psychosocial costs related to family members’ suffering; Quality of life lost providing care to family members
Healthcare system (public and private)	None
Business/industry/employers (includes government employers)	Employee morale
Government (excluding health care system)	Deadweight loss of additional taxation
**Appropriateness**	
Does the study include the relevant costs?	1 = the costs included are relevant for the objective of the stud. (minimum of 80% of the costs included in the section costs of this table)
	0,5 = the inclusion of the costs is partial
	0 = there are missing a large number of costs that should be included or there is no specification of the costs included
Are the inclusion of the costs appropriate for the objective of the study?	1 = considering the aim, all the necessary type of costs are included. (for ex for the evaluation of direct costs of a drug treatment all the costs borne by the patients directly and by the health care are included)
	0,5 = Only partial relevant costs are included. There are missing of some important costs related to the aim of the study.
	0 = Although the study aim is to consider a general costs of diabetes or a costs of drug or complications there are included only a category of costs (for ex direct costs).
Has the Diabetes severity Index been used?	1 = Yes
	0 = No
Is adequate documentation and justification given for cost components, data and sources, assumptions and methods?	1 = detailed justifications are given for all the approach and methods adopted. The exclusion and inclusion of categories of cost and data are well motivated. All the sources are documented.
	0,5 = partial justification is given for the methods and approach adopted. There is limited or absence of justifications for the inclusion or exclusion of costs. The documentation is scarce and not precise.
	0 = absence or minimal presence of documentation and justification
Are important limitations discussed regarding the cost components, data, assumptions and methods?	1 = all the most important limitations are discussed. In same cases some minor limitation is discussed.
	0,5 = one or only not important limitations are discussed.
	0 = there is no discussion around the limitations of the study.
**METHODS**	
Which is the epidemiological approach employed?	**A) Prevalence-based:** estimates the total cost of a disease in a given population for a given period. (Static)
	**B) Incidence-based:** estimates the potential averted costs if new (incident) cases are prevented. (Dynamic)
Is the data representative of the study population?	1 = prevalence-based
	0, 5 = Incidence based
	0 = no definition of the approach considered
Which approach in quantification of the costs were used?	**A) Top-down approach** refers to aggregate data available at national level, and involves a process of relating the overall health care spending to the individual diseases. From a methodological point of view to estimate the costs with the top-down method is crucial an excellent databases.
	**B) The bottom-up approach** refers to the direct consumption of resources, including epidemiological data, the cost of individual factors and the costs by the product, the average consumption of resources and its price/cost.
Was the approach appropriate?	1 = bottom-up approach.
	0,5 = top down.
	0 = no approach defined/ or impossibility to infer the approach employed
Which is the method used to evaluate the value of health?	A) Human Capital approach (generally recommended)
	B) Friction Costs approach.
	C) Willingness to pay.
How is used the discount?	Discounting is an economic method that captures an individual’s preference for income today rather than income in the future. This time preference is often explained by the opportunity-cost of interest. Income earned today can earn interest through investment.
Is the approach appropriate?	Discounting is relevant for direct and indirect costs and health outcomes that accrue past the first year.
How are estimated the costs and health outcomes ?	**A) Total disease costs:** estimating of the total health-care expenditure of people diagnosed with diabetes.
	**a) Sum_All Medical costs:** Identify all patients with a diagnosis and sum costs
	**b)Sum_Diagnosis Specific:** Identify all patients with a primary diagnosis and sum costs for treatments for that diagnosis
	**B) Incremental costs:** estimating the increase in costs that is attributable solely to the presence of the diabetes:
	**a) Matched Control:** Identify all patients with a diagnosis and sum cost. Subtract out the average cost of the sample to find incremental costs for treatment; alternatively, subtract out the average cost of a matched cohort instead
	**b) Regression_Method:** Identify all patients with a diagnosis, complete a regression analysis and indicate the individual β for each diagnosis
	Identify all patients with a diagnosis, find a matched cohort (similar to a clinical trial) and complete a regression analysis to quantify the individual β for each diagnosis – gold standard
Is the estimation method of the cost of diabetes appropriate?	1 = Incremental costs method.
	0,5 = Total disease costs
	0 = no methods designed or impossibility to retrieved a clear method from the study.
Are the deviation standard and the means calculated?	1 = both, standard deviation and Means are calculated. 0,5: only one of them is calculated. 0: none of them is calculated
Is a sensitivity analysis performed?	1 = the sensitivity analysis is performed and the results are clearly shown.
	0,5 = some linear regression method are employed to correlate the variables
	0 = no sensitivity analysis or linear regression are performed.
If yes, is it performed on:	1) Important (uncertain) parameter estimates
	2) Key assumptions
	3) Point estimates
Which statistical methods are used	1 = the statistical analysis is performed with consistent statistical formulas. The formulas used should non-parametrical statistical hypothesis test.
	0,5 = the statistical analysis is performed but only with few statistical tools.
	0 = no statistical methods are used.

### Findings

**Figure 1:**
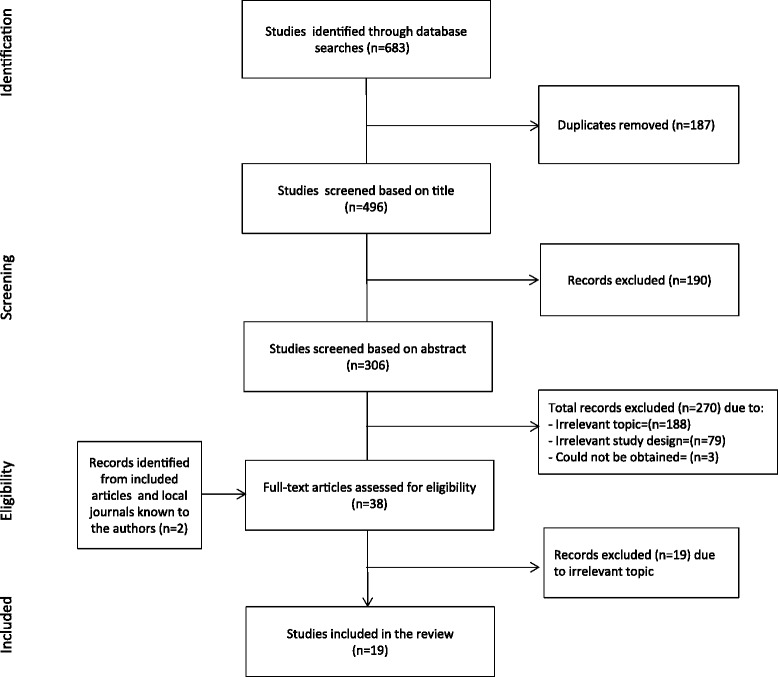
**Flow chart of the study selection process.**

A total of nineteen studies met the inclusion criteria. The flow of information through the different phases of the review is depicted in Figure 1.

A summary of the main features of the studies included is presented in Table 4. Eighteen studies were observational studies of which twelve were cross-sectional, four were cohort longitudinal and two were case control studies. Only one study was a RCT.

**Table 4:** **Study characteristics of included articles**

**Ref**	**Author**	**Year**	**Study design**	**Diabetes type**	**Type of complication**	**Sample size**	**Data collection period**	**Region**
[28]	Abdi et al.	2012	RCT	1 and 2		350	***	Southern India
[29]	Adiga et al.	2010	CS	2		238	2008	Karnataka
[30]	Bjork et al.	2003	CS	1 and 2		5516	Jan-Sep 1999	National
[31]	Grover et al.	2005	COH	1 and 2		50	***	Northern India
[32]	Joshi et al.	2013	CS	2		166	Feb-Apr 2010	Punjab
[33]	Kuchake et al.	2010	CS	2		163	Jul 2009- Feb 2010	Maharashtra
[34]	Kumar et al.	2008	COH	2		819	2005	Delhi
[35]	Kumpatla et al.	2013	CC	2		368	Jun 2008-dec 2009	Chennai
[36]	Ramachandran et al.	2007	CS	2		556	1998 - 2005	7 states
[37]	Rao et al.	2011	CS	**		1858	Jan - Jun 2004	National
[38]	Rayappa et al.	1999	CS	1 and 2		611	1997 - 1998	Bangalore
[39]	Shivaprakash et al.	2012	COH	1 and 2		200	2005 and 2010	Mangalore
[40]	Shobhana et al.	2000	CC	2		270	Jan - Jun 1998	Chennai
[41]	Shobhana et al.	2002	CS	1		209	Jan - Oct 2000	Chennai
[42]	Shobhana et al.	2000	CS	2		596	1999	Chennai
[43]	Tharkar et al.	2010	CS	2*		718	Aug -Dec 2009	Chennai
[44]	Akari et al.	2013	COH	1 and 2		150	Feb-July 2012	Hanamkonda
[45]	Satyavany et al.	2014	CS	2		209	Aug 2008- Jan 2010	**
[46]	Tharkar et al.	2009	CS	2		443	Oct- Dec 2007	**

CC = Case Control Study CS = Cross Sectional Study, COH = cohort study, RCT = randomised controlled trial.

*The study does not clearly state whether it refers to diabetes type 1, type 2 or both. We assume type 2 diabetes as the articles references refer to type 2 diabetes studies.

**The information is not provided in the study.

***Year of data collection could not be identified. Year of publication utilised as proxy.

**Figure 2:**
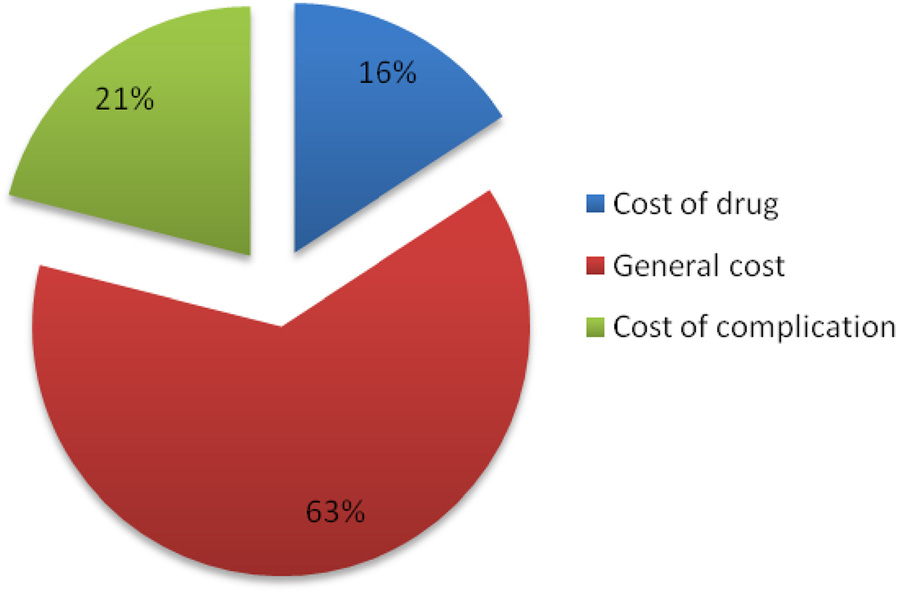
**Study objective.**

Sixty-three percent of the studies dealt with the general costs of diabetes while 21% focused only on diabetes complications, including diabetic foot wound (DFW) and chronic kidney disease, and 16% of the studies analysed the cost of a specific drug for the treatment of diabetes (Figure 2).

The study samples varied from 50 to 5,516 individuals, and from local, regional, cross-regional to national studies. A summary of the studies reviewed is presented in Table 5.

**Table 5:** **Main cost data of reviewed studies**

**Ref**	**Author**	**Year of data**	**INR per USD**	**Health system costs INR (USD current value/USD 2014 value)/person**		**Individual/household costs INR (USD current value/USD 2014 value)/person**	
				***Direct***	***Indirect***	***Direct***	***Indirect***
[28]	Abdi et al.	2012*	53.06	Drug consumption (DDD^1^/100 bed days) 13.42 (0.25) (0.26)			
[29]	Adiga et al.	2008	39.41			Annual costs of consultations and drugs 19,076.07(484.04/535.14)	
[44]	Akari et al.	2012	53.06	Average cost for patient with diabetes complication (including costs of drugs, consultations, hospitalisation, (314.15/325.69), without (29.91/30.17)			
[30]	Bjork et al.	1999	42.49			Total costs, annual (drugs, monitoring, check-ups, hospitalisation) 7,159 (168.49/240.73)	
[31]	Grover et al.	2005*	43.40	Total costs over 6 months (incl. consultations, investigations, nursing, infrastructure) 205.55(4.74/5.78)		Total costs over 6 months (incl. drugs, food, travel) 4,966.42 (114.43/139.47)	Total costs (income loss, patient and caregiver) 2,086.74(48.08/58.60)
[32]	Joshi et al.	2010	46.61			Cost per consultation 166 (3.56/3.89)	
[33]	Kuchake et al.	2010	46.61			Cost per consultation 116.85 (2.51/2.74)	
[34]	Kumar et al.	2005	43.40			Total costs per year (incl. consultations, tests, drugs, monitoring) 6,212.4 (143.14/174.76)^2^	
[35]	Kumpatla et al.	2009	48.76			Total cost without complication 4,493 (92.15/102.24), with complication(s) 15,280 (313.37/347.69)^3^	
[36]	Ramachandran et al.	2005	43.4			Total costs (incl. drugs, tests, consultations, hospitalisation, surgery, median) 8,130 (187.33/228.32)^5^	
[37]	Rao et al.	2004	45.60			Costs per hospitalisation 5925(136.5/172)	
[38]	Rayappa et al.	1999	39.10	Annual societal costs (incl. routine, monitoring, tests, hospital) 1,305.20 (33.38/47.69)	Annual societal costs 15,376.30 (393.25/561.86)	Annual costs (incl. routine, monitoring, tests, hospital) 15460.40 (395.41/564.95)	Annual costs 3,572.5 (91.37/130.55)
[45]	Satyavany et al.	2010	46.61			Total annual costs for a patient with Kidney problems associated with diabetes: 392,920 (8450/9224.06)[transplantation]; 61,170 (1,315/1435.46) [dialysis]; 12,664(272296.62)[CKD stages]; 3,214 (69/75.32)[without complications]	
[39]	Shivaprakash et al.	2005	43.40			Cost per consultation 363(8.36/10.19)	
[40]	Shobhana et al.	1999	39.1			Costs during 6 months (incl. consultation, surgery, hospitalisation, tests, drugs, transport)12,055(308.31/440.50^6^	
[41]	Shobhana et al.	2000	Values for both currencies as provided in the article			Total annual costs (including drugs, tests, consultation, hospital, transport) Inpatient 15,596(331.8/468.65), Outpatient 8,578(200.7/277.3)	
[42]	Shobhana et al.	1999	42.49			Total annual costs (incl. drugs, tests, consultation, hospital, surgery, transport) private hospital 4,510 (106.1/151.59), public hospital 246 (5.8/8.29)	
[43]	Tharkar et al.	2009	48.76			Total annual costs (incl. consultation, drugs, investigation, transport, food, miscellaneous, accommodation for alternate caregiver, management) 25,391 (520.73/577.76)	
[46]	Tharkar et al.	2007	44.11			Total costs hospital admission during 2 years (Incl. drugs, investigations, miscellaneous, admin), without comorbidities 28,000 (634.78/728.73), with comorbidities 38,000 (861.48/988.99)	

Notes: USD current refers to the value reported in the study, USD 2014 it the value adjusted to 2014 price levels using http://www.bls.gov/data/inflation_calculator.htm.

CKD: Chronic kidney disease; INR: Indian rupee.

*Year of data collection could not be identified. Year of publication utilised as proxy.

^1^DDD: defined daily dose.

^2^ Total costs per year are averaged between male and female.

^3^Values for patients with complications are average of 5 groups: renal, cardiovascular, foot, retinal, two complications.

^4^Values are average of treatment arms: human insulin and blood, glucose monitoring, bovine insulin and blood glucose monitoring, bovine insulin and urine glucose monitoring.

^5^Values are average of urban and rural population.

^6^Values are average of outpatients and hospital patients with foot problems.

**Figure 3:**
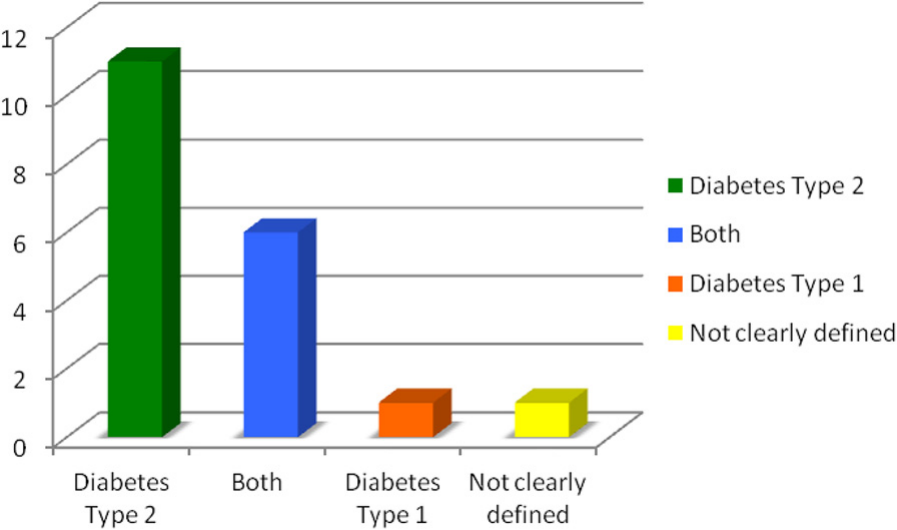
**Type of diabetes considered.**

With regards to the type of diabetes analysed, most studies (n =11) considered the cost of diabetes mellitus type 2, six studies considered the costs of both, only one study focused on the cost of diabetes mellitus type 1 and one study did not clearly define the type of diabetes considered (Figure 3).

### Different types and perspectives of costs

**Figure 4:**
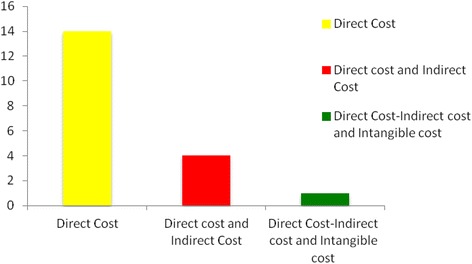
**Costs included.**

Overall, the majority of the studies included only direct costs in their evaluation (n =14), 4 studies included direct and indirect costs and only one study included direct, indirect and intangible costs (Figure 4).

Most studies (17 studies) report on the costs to the individual, while only two studies report on costs for the health system.

#### Health system perspective

Both studies using a health system perspective reported costs for consultations and medicines [31,38] and drug costs [31,38]. Studies reported that the costs to hospitals and other health providers constituted only a small part of total diabetes costs. In the study on ambulatory diabetes care in northern India, the authors found that the mean cost borne by the hospital over a six-month period was 2.83% of the total direct costs. No study reflected on indirect costs from a societal perspective, although one study provided annual societal indirect costs at INR 15,376.30 (USD 393.25) [38].

#### Direct costs

Direct costs were investigated in all the reviewed studies. Detailed costing data for these studies are provided in Table 6. The most common cost item reported on was drug costs (12 studies), followed by hospital related costs (11 studies), consultation costs (11 studies), laboratory costs (10 studies) and transport costs. Less common cost items were surgery costs (3 studies), monitoring costs (2 studies) and food costs (2 studies). In six studies providing estimates for cost components as well as total costs, drug costs accounted for more than half of the total direct costs [31,34,36,40,41,43,47]. A study from Delhi reported that the average annual direct cost of type 2 diabetes was INR 6,212.4 (USD 143.14) in 2005, of which more than half were drug costs (INR 3,324; USD 76.59) [34]. Similarly, a study from northern India on diabetes type 1 and 2 reported a total direct cost of INR 4,966 (USD 114.4) over six months in 2005^a^; 62% of the total direct cost were drug costs (INR 3,076; USD 70.88) [31] Table 6.

**Table 6:** **Costing items and estimates per person in studies reporting on direct cost of diabetes for individuals and households INR (USD current value)**

**Ref**	**Drug**	**Test/investigation**	**Monitoring**	**Transport**	**Hospitalisation**	**Consultation**	**Surgery**	**Food**
[33]						Cost per consultation 116.85 (2.51)		
[34]	Costs per year 3,324.45 (76.60)	Costs per year 1803.35 (41.55)	Costs per year 322.75 (7.44)			Costs per year 875.85 (20.18)^2^		
[35]	Costs without complications 800 (8.20), with complications 1,960 (40.20)^3^			Costs without complications 300(6.15), with complications 830 (17.02)^3^	Hospital charges without complications 1,083 (22.21), with complications 5,256.4 (107.80)^3^	Costs without complications 350 (7.18), with complications 1,085 (22.25)^3^		
[36]	Median costs at 3,250 (74.88)^6^	Median costs at 1,000 (23.04)^6^			Median costs at 8,000 (184.33)^6^	Median costs at 800 (18.43)^6^	Median costs 13,750 (316.82)^6^	
[37]					Costs per hospitalisation 5,925 (136.5)			
[29]	Annual costs, 18,623.94 (472.57)					Annual costs, 452.13 (11.47)		
[38]			Annual monitoring and lab costs 822.6 (21.04)		Annual costs 8,678.6 (221.96)			
[39]						Cost per consultation 363(8.36)		
[40]	Costs during 6 months 3,000 (76.7)^7^	Costs during 6 months 1,435 (36.70)^7^		Costs during 6 months 225 (5.75)^7^	Costs during 6 months 3,650 (93.4)^7^	Costs during 6 months 1,900 (48.59)^7^		
[41]	Annual costs Inpatient 6,840 (145.5), Outpatient 6,150 (130.8)	Annual costs Inpatient 630 (13.4), Outpatient 400 (8.4)			Annual costs 5,000 (106.3)	Annual GP and specialist costs Inpatient 550 (11.5), Outpatient 420 (8.8)		
[42]	Annual costs, private hospital 3,000 (70.6), public 735 (17.3)	Annual costs, private hospital 360 (8.5), public hospital 240 (5.6)		Annual costs, private hospital 240 (5.6), public hospital 192 (4.5)	Annual costs private hospital 5,000 (117.7), public hospital 0.0	Annual GP and specialist costs, private hospital 600 (14.12), public hospital 670 (15.7)	Annual costs private hospital 9,000 (211.8)	
[43]	Annual costs Hospital 1,500 (30.76)	Annual costs, hospital 2,250 (46.14), ambulatory 1,050 (21.53)		Annual costs, hospital 600 (12.30), ambulatory 202 (4.14)		Annual costs, hospital 550 (10.37), ambulatory 320 (6.56)		Annual costs, hospital 600 (12.30), ambulatory 190 (3.89)
[46]	Average cost per hospitalisation, without comorb. 184 (4.17), without comorb. 2,098 (47.56), outpatient without comorb. 456 (10.34), with comorb. 488 (11.06)	Average cost per hospitalisation, without comorb 903 (20.47), with comorb 968 (21.94), outpatient without comorb. 373 (8.46), with comorb. 405 (9.18)			Cost per hospital admission, without comorb. 18650 (422.80), with comorb. 2,1000 (476.08)			
[45]	Transplantation 40,400(869); dialysis 7250 (156); CKD 1,500 (156); No complications 800 (17)	Transplantation 64,925 (1.396); dialysis 2800 (60); CKD 3,625 (78); No complications 1,214 (26)		Transplantation 3,250 (70); dialysis 3480 (75); CKD 625(13); No complications 300(6)	Transplantation 21,5000(4,624); dialysis 22,000 (473); CKD 4,010(86);No complications 1,082(23)	Transplantation 67,000 (1,441); dialysis 32,200 (692); CKD 1,000 (121); No complications 350 (8)	Total cost for Transplantation 392,920 (8,450)	
[44]	Average per patients: Without complications 380 (7) with complications 3,769 (69)	Average per patient 1,598 (29.45)			Average cost per patient 7,800 (143.75)	Average cost per patient 2,191 (40.37)		
[31]	Costs during 6 months 3,076.28 (67.46)	Costs during 6 months 277.80 (6.09)		Costs during 6 months 458.96 (10.04)				Costs during 6 months 72.66 (1.59)
[30]					Annual costs 2,435 (57.31)			
Totall^1^	12	10	2	6	11	11	3	2

^1^Total number of studies addressing the costing item.

^2^Values are averages between male and female.

^3^Values for patients with complications are average of 5 groups: renal, cardiovascular, foot, retinal, two complications.

^4^Values are average of treatment arms: human insulin and blood, glucose monitoring, bovine insulin and blood glucose monitoring, bovine insulin and urine glucose monitoring.

^5^Values are average of treatment groups: teleophthalmology (screening), hospital (dilated retinal examination), hospital (laser photocoagulation). We assume costs are yearly estimates.

^6^Values are average of urban and rural population.

^7^Values are average of outpatients and hospital patients with foot problems.

#### Indirect costs

Indirect costs of diabetes and its complications were reported in four studies. A study from northern India reported a total INR 2,087 (USD 48.09) indirect costs over a six-month period in 2005^a^. Patient income loss accounted for 61% of the total indirect cost (INR 1,263, USD 29.10) while the remainder 39% (INR 823, USD 18.96) was due to income loss of the carer [31].

#### Socioeconomic burden of diabetes

Several studies investigated differences in costs as related to one or several demographic and socioeconomic parameters by looking at levels of income, education and occupational status, and by comparing costs in rural and urban populations [30,31,34,36,43,48]. Several studies found that lower income groups generally spent a larger proportion of their income on diabetes care, that urban populations spent more in absolute terms, and that cost of complications weighed heavily on overall costs.

Within the diabetes population, low income individuals bear the highest burden of diabetes [40]. A study on type 2 diabetes in seven states in India during the period 1998 to 2005 found spending to be higher among the urban than the rural population both in absolute terms and as a proportion of income. This was due to higher expenditure on medical consultations, laboratory tests and drugs, which the authors attributed to the use of more expensive treatments in urban areas (which have remained unavailable in rural areas). Also, in lower-income groups spending was higher in the urban than the rural population, possibly because awareness of diabetes care was better among the urban poor [36]. A Chennai-based study in 1999 compared costs for type 2 diabetes in public and private institutions and found that individuals seeking care in private hospitals were economically better off, and that families who could afford it preferred private provision over state-funded care as the public hospitals were crowded and the staff overworked [42]. A study from Bangalore with cost data from 1997 and 1998 found that uneducated, unemployed people in semi-urban or rural areas were more likely to be diagnosed later as they could not afford to consult a doctor, and therefore developed complications [38]. Treatment costs were found to be significantly higher in those who were more educated in a study from northern India [43]. Patients with less than five year of education spent INR 398.66 (USD 9.19), while those with more than five years education spent INR 2,810.20 (USD 64.77).

#### Complications

**Figure 5:**
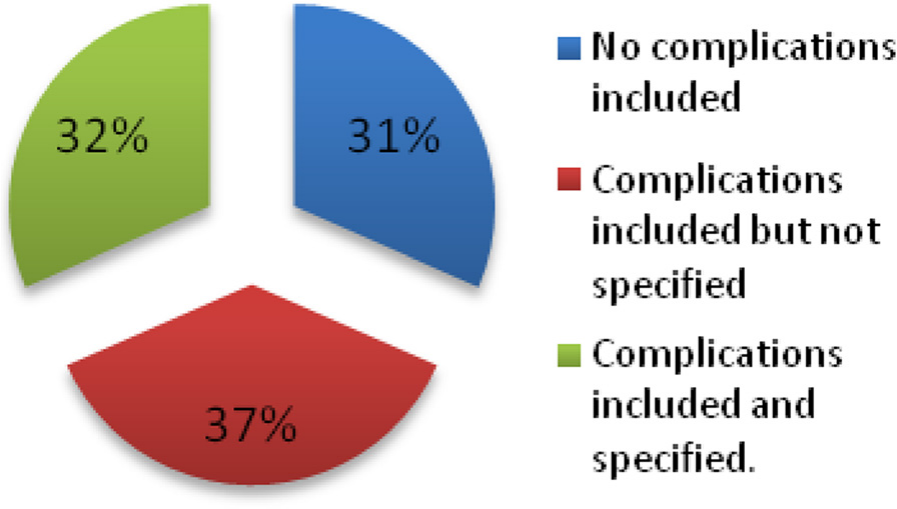
**Complications included.**

Sixty-nine percent of the studies included complications in their evaluation of the cost of diabetes. Only 32% of the studies [29,33,40,45,46] have specified the type of complications included while 37% of the studies only identified the presence of a number of complications (1 to 3) without specifying the type (Figure 5).

Studies considering diabetes complications indicated that they weighed heavily on the overall costs. For example, the number of complications per patient was found to be positively correlated with the patient’s healthcare expenditure [30,36]. However, no significant urban/rural differences were found in the prevalence of complications of diabetes [36]. Studies argued that any measure to reduce hospitalisation costs would sharply reduce the economic burden for households and society, and increase patients’ quality of life [30]. Further, that substantial cost savings could be achieved by focusing on provision of care in outpatient settings [40].

Two studies compared costs of diabetes care for patients with and without complications [35,46]. A study from Chennai reporting on costs from 2008 and 2009 found that total costs for patients without complications were INR 4,493 (USD 92.15) compared to INR 14,691.75 (USD 301.32) for patients with complications^b^ [35]. Among the different types of complications investigated, foot complications incurred the highest costs; patients with foot complications spent four times more than patients with no complications. Patients with renal disease, cardiovascular and retinal complications spent three times more than those without complications. Consultation and hospitalisation costs were especially high for patients with complications (on average INR 1,085 (USD 22.25) for consultation costs and INR 5,256.4 (USD 107.80) for hospital costs compared to patients without complications INR 350 (USD 7.18) for consultation costs and INR 1,083 (USD 22.21).

#### Quality analysis

The analysis focused on the key elements necessary to perform a good cost of illness analysis. Most of the studies (n =11) scored less than 10 on 17 points scale. Interestingly, the remaining 8 studies reached a score slightly higher, with a maximum score of 13.5. The median score was 9.5.

Overall studies lacked an accurate and precise definition of the disease, with only 4 articles referring to WHO definition of diabetes, and only 3 studies gave a clear definition of the type of diabetes considered.

Most studies developed their research over an adequate period, usually of 6 months, while two studies did not specify the timeframe.

Although we considered discounting in the qualitative table, we have not accounted for it as a quality element for two main reasons. First, the prevalence-based studies considered a time short-term horizon and the need to apply a discount rate is the subject of an on-going debate [27]. Second, for incidence-based studies, the appropriate approach for calculating the discount is still an unsettled matter in the literature [49].

The majority of the studies (84%) considered an appropriate number of patients or household for the purpose of their study objective. The benchmark employed is based on the work of Krathwohl, which provides a number of questions to individuate if the sample is appropriate in comparison with the purpose of the study [50].

The remaining 16% of the studies consider samples that either are too small or do not state the size of the sample considered. Further, it is important to note that the majority of the studies only considered the middle and high-income portion of the Indian population due to the difficulties involved in collecting data on the low-income classes.

All studies used a questionnaire, or a survey, to collect the data based on self-assessment of illness and costs. In addition, 12 out of 19 studies validated the reliability of the self-assessment against hospital bills and clinical records retrieved directly from the hospitals or practitioners.

The second part of the quality analysis considered the appropriateness of the various types of costs that each study included. The appropriateness of cost inclusion was benchmarked against the study objectives and the minimum requirements for a sound cost of illness study according to international best practice [27,51]. Only 52% of the studies included the appropriate costs, both in terms of their objective and in terms of minimal requirements for a sound cost of illness analysis. In one case, it was not possible to assess the relevance and the appropriateness of the costs included due to a lack of information on data sources and categories of cost.

**Figure 6:**
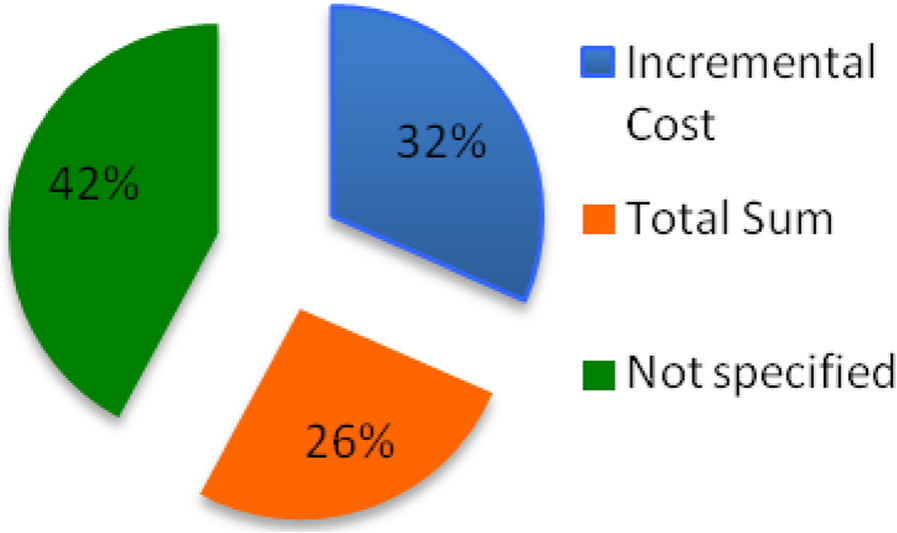
**Cost estimation methods.**

In terms of methods, most studies lacked sufficient details on the methods used. In particular, 42% of the studies did not specify how costs were estimated. Only 32% of the studies adopted the incremental costs method, which is the most appropriate for diabetes, and only 4 studies mentioned the use of either matched control (n =2) or regression method [24] (Figure 6).

Results indicate that the prevalence-based approach, with a bottom-up quantification of the costs, was the most common method used to conduct cost of diabetes studies in India. Notably, 16 studies employed a prevalence-based approach and measured diabetes attributable costs that occurred concurrently with prevalent cases over a specified time period, usually 6 months (Figure 6).

A bottom-up approach was used in 15 studies by assigning costs to individuals with diabetes based on clinical practice data.

Regarding the evaluation of uncertainty, the majority of the studies did not perform any type of analysis. In fact, only one study performed a sensitivity analysis and 3 studies conducted linear or multivariate regressions.

**Figure 7:**
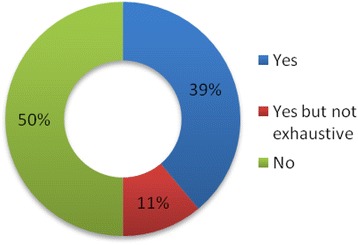
**Limitations discussed.**

In addition to inconsistencies regarding the type and extent of information provided on methods, a discussion of limitations was largely absent (Figure 7). 50% of the studies did not mention any limitation, while 11% mentioned only one minor limitation, such as related to the size of the sample (n =2). Only 39% of the studies provided a comprehensive discussion of the limitations of the cost components, data, assumptions and research methods.

Regarding the statistical methods employed, 14 studies performed the necessary statistical analysis for a good quality study. The majority employed the student t-test to determine the statistical significance and the Wilcoxon matched pair signed-rank test to verify the validity of the data. A number of studies employed the Chi-square test and percentage value to validate their data. A large number of studies used the statistical package SPSS to analyse the data.

Two studies state the presence of statistical analysis. However, they did not identify which statistical formulas had been used. One study even declared that it had not performed any kind of any statistical analysis at all.

11 studies presented the standard deviation along with the mean estimate while 4 studies included only the mean.

### Discussion and recommendations

With the population of people living with diabetes predicted to rise above 109 million by 2035 [17], there is an urgent need to act at all levels of authority in India, and with additional coordination at the national level. Further, there are several specific areas in which policy makers could concentrate efforts to reduce the impact of the economic burden of disease.

Firstly, the economic burden falls heavily on patients and their families and requires better health care coverage. There is a need to mitigate the serious adverse effects of high out-of-pocket expenditure, including impoverishment of catastrophic spending and cost of complications. To this end, efforts, such as the expert group set up by the Planning Commission of India to achieve universal health coverage by 2022 [52] need to be considered in order to increase coverage and pool healthcare costs across the population. Policies aiming to strengthen health systems are also essential in this process.

Secondly, high costs and suboptimal access to drugs contribute significantly to the burden of the disease and should be addressed through market shaping strategies. While hospitalisation and complications are major components of the costs of diabetes, drug costs constitute an important part of the expenses, often representing more than 50% of total direct costs for households. A study based on a large dataset, found that drug costs accounted for 58% of out–of-pocket expenditure on diabetes [53]. Another study on drug costs as share of expenses paid out of pocket by quintile group revealed progressive private spending on health, with the poorest spending 75.42 percent on drugs, compared to 65.9 percent spent on drugs by the richest in 2009–10 [12]. By further comparison, studies of diabetes in Western countries shows that drug costs constitute a much lower share of total direct health expenditure on diabetes, ranging from 6.2 percent to 10.5 percent [54,55] in Europe and 12 percent in the United States [56]. In addition to better drug coverage for individuals, Indian authorities, together with the international community, should aim to employ market-shaping mechanisms to increase the access of medicines in India. Poor procurement procedures and weak supply chain systems are major barriers to access to medicines in India, contributing to low competition, low quality, high price and variable availability of drugs [12]. Pooled drug procurement of essential medicines between several Indian states has proven efficient for essential medicines [57], and should therefore be considered for medications for diabetes and related drugs.

Thirdly, lower expenditure among the rural and low income population may be due to issues of access and affordability rather than lower need [6], and late detection of the disease in these settings often leads to catastrophic spending for individuals and households [38]. Early detection and treatment provided in outpatient settings has been identified as an important means for cost reduction [30,40] and should thus be strengthened. Socioeconomic differences and the urban–rural divide suggest divergence in disease outcomes. In other words, the relatively wealthier population living in urban areas spend more on diabetes care and have better outcomes, while relatively poorer people living in rural areas tend to have more difficulties accessing diabetes care, and therefore spend less on diabetes care and tend to have worse health outcomes [58]. Mobile health units, which can increase access in remote areas, may help mitigate these socioeconomic differences.

With regards to the methodological quality of the studies considered, only a few of the studies adhered to recognised standards of methodological quality, which utilised a transparent methodology, and thus provided credible results.

The aim of COI is to identify, measure and value the resources consumed by a disease in order to determine not only the total cost, but also all the elements and methods used to design the analysis itself [24]. However, the majority of the studies failed to achieve this aim due to a lack of solid methodology.

First of all, the lack of both a clear definition and foundation in the literature, or justifications for applying new approaches, for the methods employed affect the reproducibility of the studies. Notably, the total costs were often calculated without providing a detailed list of unit costs and resource consumption was also rarely described. In addition, the majority of the studies lacked of a clear epidemiological definition of diabetes which also lead to comparability problems [59].

Secondly, the lack of a clear justification of the cost components and the data sources, together with the lack of a discussion on the intrinsic limitations of the study, produced doubts about the quality of the research. The absence of these elements could either be indicative of lack of accuracy of the study or even aimed at hiding possible gaps and/or errors in the collection of data and the calculations of costs [51].

To enhance the transparency of the cost of illness studies, it appears fundamental to provide sufficient documentation on data sources, assumptions and estimation methods [51].

In terms of costs included, there are a number of factors that could have led to possible biases in the estimation of the economic burden of diabetes in India.

One of such factor is the absence, in the majority of studies, of the cost of complication or a description of complication profile of the included patients. In particular, studies failed to include health care utilisation costs associated with chronic complications of diabetes, which are usually the most expensive [59]. Indeed, according to WHO [59] data and to a number of studies outside India [60], the treatment of patient with diabetes for other complications and comorbidities is a major source of the increasing in the health care expenditure on diabetes.

The exclusion of the estimation of the intangible costs and the loss of productivity leads to an underestimation of diabetes. Loss in productivity for the patient or carers was shown to represent up to half of the total costs of diabetes [30]. Despite difficulties in their extraction and quantification, both costs are important for a comprehensive calculation of the actual cost of the disease, which affects not only diabetes patients, but also their families and the society [25,51]. The inclusion of intangible costs is especially important in studies aiming to give a general analysis of the burden of this disease in the country or in a specific region.

In terms of perspective of analysis, the third party payer is the most common perspective adopted in the studies reviewed. The exclusion of the perspective of the healthcare sector and the households as well as the governments and local authorities excludes a number of key costs, such administrative costs and personnel costs.

The implementation of a comprehensive and accurate estimation of the cost of diabetes enables the use this cost as both a baseline and a reference, which can help to identify the programmes and strategies most effective in reducing costs associated with diabetes [50].

From a methodological perspective, most studies used a prevalence-based epidemiological approach and a bottom up quantification of the costs method, both of which are considered the most accurate and consistent for the calculation of the burden of diabetes [25,51]. Nevertheless, they also lack of other major elements for a complete COI.

The absence of an estimation of uncertainty in a large number of the studies is an important limitation. Due to the large number of uncertainties involved in a COI, it is necessary to consider alternative values for all important parameters and assumptions [50,51]. Therefore, it is necessary to conduct a proper sensitivity analysis [26,29,61].

Cost of illness studies are an important instrument for informing and raising awareness among policy-makers by providing economic information to support their decisions. Further, results of this type of economic evaluation are often used to justify the allocation of more resources to prevent and treat a certain illness [26,39]. More efforts in designing study methodologies are necessary to improve the quality of studies on the cost of diabetes in India.

Therefore, it would appear advantageous to develop and implement standardised guidelines regarding the conduct of comprehensive and accurate cost of illness studies in India. Certainly, a well designed methodology and an accurate computation and inclusion of all the costs would enhance the COI validity as a policy tool.

### Limitations

This review provides a fragmented picture of the economic burden of diabetes in India. Given the heterogeneity of study designs and diversity of methods used in the literature reviewed, we were unable to generate meaningful aggregate data for meta-analysis purposes. This heterogeneity also complicated the synthesis of the papers, and comparisons should be treated with caution due to the variability in study design and thematic focus. Future studies should aim to explore optimal methodological study designs that may facilitate the production of meaningful national estimates for meta-analysis.

## Conclusion

This study has aimed to inform the discussion on the economic burden of diabetes by reviewing the literature on diabetes costs for individuals and society. We found that most studies on the costs of diabetes and its complications in India have focused on the costs borne by patients, both direct and indirect, and less evidence exist on the economic burden for the health care system and society. Three areas of concern were identified for policy interventions. First, the heavy economic burden of diabetes borne by individuals should be reduced via the improvement of universal healthcare coverage. Second, market shaping mechanisms should be considered to improve the access to affordable medicines, which constitutes an important part of private costs. Finally, early disease detection and treatments in outpatient settings provide cost saving ways of tackling the disease.

As the epidemiological burden of diabetes increases, the economic burden on households is expected to rise and the economically disadvantaged will be the most affected. Future initiatives to tackle diabetes type 1 and 2 should be grounded in evidence-based and integrated strategies of prevention and disease management, and implemented at all levels of authority. Cost of illness analysis should be a basis on which strategies for mitigating the effects of this pervasive illness gain a higher priority on the health policy agenda.

## Endnotes

^a^The authors do not provide the year of data collection and the year of article publication is used as a proxy.

^b^Values are averaged across the different types of complications: renal, cardiovascular, foot, retinal.

## Abbreviations

CRF: Chronic renal failure
CKD: Chronic kidney disease
DALYs: Disability adjusted life years
GDP: Gross domestic product
KT: Kidney transplant
DFW: Diabetic foot wound
INR: Indian rupee
NCDs: Non-communicable diseases
PRISMA: Preferred reporting items for systematic reviews and meta-analyses
RCT: Randomised controlled trials
WHO: World health organization
